# Predictors and Timing of ATT Initiation among HIV-TB Patients at ART Centers of Karnataka, India: Two Year Follow-Up

**DOI:** 10.1371/journal.pone.0138603

**Published:** 2015-09-22

**Authors:** Suresh Shastri, Sharath Burugina Nagaraja, Jaya Prasad Tripathy, Srinath Satyanarayana, Bharat Bhushan Rewari

**Affiliations:** 1 State TB office, Bangalore, Karnataka, India; 2 ESIC Medical College and PGIMSR, Bangalore, Karnataka, India; 3 International Union Against Tuberculosis and Lung Disease (The Union), South East Asia Office Regional Office, New Delhi, India; 4 National AIDS Control Organization, New Delhi, India; University of Delhi, INDIA

## Abstract

**Background:**

In India, TB and HIV co-infection remains as a serious public health problem. From 2006 onwards, the intensified TB-HIV collaborative activities are being jointly implemented by National AIDS Control Programme (NACP) and Revised National TB Control programme (RNTCP) at high HIV burden states.

**Objectives:**

To determine (a) the predictors of outcome among a cohort of HIV-TB co-infected patients after two years after initiation of ART treatment. (b) prognostic significance of time difference between the initiation of ATT and ART in HIV-TB co-infected patients.

**Methods:**

Patients registered at sixteen ART centres in Karnataka, from October through December 2009 formed the study cohort and were followed till December 2011.

**Results:**

A total of 604 HIV-TB patients were registered. Follow-up (a) at the end of one year had shown 63.6% (377)patients with unfavorable TB treatment outcomes (b) at the end of second year, 55.6% (336)patients were alive on ART treatment. The variables male, smear negative TB, CD4 count less than 50cells per cumm and unfavorable TB outcome were significantly associated with unfavorable ART treatment outcome.

**Conclusions:**

The programmes need to review the existing strategies and strengthen HIV-TB collaborative activities for timely treatment initiation with intensive monitoring of HIV-TB patients on treatment.

## Introduction

In high TB burden countries like India, tuberculosis and HIV pose to be a serious public health problem. HIV augments the progression of latent TB to active TB, relapse and death [1]. Certainly, TB is the commonest cause of mortality among people living with HIV (PLHIV).[2] India accounts for 10% of total HIV-TB cases globally. [3] To combat and control the co-infection a strategy of intensified case finding is adopted by the country. It includes the routine screening of TB patients for HIV at integrated counseling and testing centres (ICTCs) and anti-retroviral centres (ART). For those patients who are reactive to HIV- care, support and treatment is provided at ART centres. Since 2011, the National TB-HIV collaborative activities are implemented vigorously to enhance early initiation of ART in HIV infected TB patients.

Though, systems are in place to provide ART services for diagnosed HIV-TB patients, there is a substantial gap in the knowledge about socio-demographic and clinical factors associated with poor treatment outcomes. The relevance to bridge this gap is extremely important as it drives the programme to adopt suitable strategies that vouch for better treatment outcomes. Hence, we conducted this study to determine the outcomes and explore the predictors for outcome among a cohort of HIV-TB co-infected patients registered at ART centers.

Though, many prospective studies have evaluated the optimal time for initiating ART in HIV-TB co-infected patients and have demonstrated reduced mortality among early ART initiators;[4] there lacks a clarity about the timing for ART initiation in HIV-TB co-infected patients that impacts clinical outcomes under routine programme settings. We intend to study the effects of time difference between the initiation of ATT and ART on all-cause mortality in a cohort of HIV-TB co-infected patients.

## Methodology

A retrospective cohort study was conducted in the state of Karnataka, south India. The state of Karnataka is one of the high HIV burden state in the country with adult HIV prevalence exceeding 1% in some districts of the state.[1] The intensified TB-HIV collaborative activities that includes screening of all TB patients for HIV and screening all HIV patients for TB is being implemented in the state from 2006 onwards. All patients found to be HIV-TB co-infected are referred to ART centre where the CD4 cell count are evaluated. Patients with CD4 count below 350 cells per cumm, were initiated on ART treatment else they were given cotrimoxazole prophylaxis therapy (CPT) and monitored at regular intervals. All the HIV-TB co-infected patients eligible for ART were enrolled and treatment was provided regularly on a monthly basis for their lifetime.

To ensure smooth implementation of the Revised National TB Control Programme (RNTCP) the Karnataka state is divided into 129 tuberculosis units (one tuberculosis unit is the functional reporting unit of RNTCP for every 0.5 million population) with 641 Designated Microscopy Centers (DMC) (the sputum microscopy centre for 0.1 million population). The National AIDS Control Programme (NACP) has 565 Integrated Testing and Counseling Centres (ICTCs), 1050 facility integrated ICTCs (FICTCs) and 49 ART centers in the state. These centres maintain the details of patients undergoing HIV counseling and testing; those found to be HIV positive are referred to the nearest ART center for further evaluation and management. At ART centres, patients are registered and then undergo basic investigations including CD4 count; if found eligible according to the NACP guidelines they were initiated on treatment. During the study period, ART centers followed the 2009 NACP guidelines for treatment initiation.[3]

### Study population, sampling and sample size

The study was conducted at 16 ART centres of Karnataka, south India. The ART centres were selected randomly from four geographical regions of the state. Centres which were established before 2007 and with established referral mechanisms were selected for the study. All HIV-TB patients registered during the period October to December 2009 were included in the study and were followed up to two years from the point of treatment initiation i.e., till December 2011. There were 704 eligible HIV-TB patients registered and initiated on ART at these centers. Based on the completeness of the records maintained in the registers 604 patients were included in the study.

### Sources of data and data analysis

The details of patients initiated on treatment were collected from the ART registers at ART centres. The ART registers are regularly updated by the ART staff. Death ascertainment was done through routine monthly field visits by the ART staff with the help of local health workers. The health care workers during their routine home visits enquire about the cause of death and report it to the medical officer who confirms the cause of death. The TB registers were also referred to validate the TB cases in ART registers. Variables like age, sex, type of TB treatment, category of TB treatment, date of starting ART treatment, date of starting anti-TB treatment (ATT), CD4 cell count and treatment outcome of the patient were collected in a data collection proforma by the trained health workers.The data entry was done in Microsoft excel sheet and the data was analyzed using Epi-info (version 3.5.4) and Epi-data software (version 2.2.2.182). Chi-square test was used to look at the association of socio-demographic and clinical variables with treatment outcomes. Multivariate logistic regression was used to determine the predictors of unfavorable outcomes after adjusting for confounder variables. Survival probability by month of treatment was determined by the Kaplan-Meier method. Cox proportional regression was used to calculate Hazard Ratios for mortality.

### Operational definitions

“Favourable TB treatment outcomes” included patients who were cured or treatment completed while “Unfavourable TB treatment outcomes” included those who had failed treatment, loss to follow-up, died and transferred out while on treatment. “Favourable ART treatment” included patients who were alive and on ART while “Unfavourable ART treatment” included those died, loss to follow-up, migrated, stopped treatment or transferred out while on treatment.

Our study is an operational research study based on the review of existing records and reports under programmatic settings. Hence, we did not seek Institutional Ethics approval. The permission from the concerned higher authorities like the Karnataka State AIDS Prevention society (KSAPS) and State TB cell, Karnataka was obtained for the conduct of the study. As per the NACP policy, all patients attending ICTCs are counseled by professionally trained counselors, and informed consent for the same is obtained from the patients during the first counseling. NACP field staff routinely follows the HIV patients on regular basis. In this study, the records and reports from the HIV registers and treatment cards maintained at the ART centres and the specific data focusing the study objectives were analysed. All patients in the register were given unique identification number and subsequently unique identification numbers were used for data analysis. The patient data was anonymized and no author involved in the study has access to identify patient information at any point of time. The participants had provided their written informed consent for HIV screening and in case of minors consent was obtained from the caretakers. All the participants had signed the consent form for the same which is as per the NACO guidelines.

## Results

A total of 704 HIV-TB co-infected patients were registered at ART centres and 604 of them with complete records were included in the study; they were followed for a period of 24 months. There were no baseline differences in key demographic and clinical variables between the patients who were included and those who were excluded from the study due to missing data. **(Table 1)**The characteristics of HIV-TB co-infected patients eligible for ART registered at 16 ART centres in the state of Karnataka during October to December 2009 are shown in Table 2. Majority of patients were males (65%), married (68%), smear positive TB (50.5%) and initiated on Category I ATT regimen (86.6%). About 64% of the patients had unfavorable TB treatment outcomes and only 10% of patients had CD4 count at registration above 350 cells per cumm. **(Table 2)**

**Table 1:** Comparison of demographic and clinical characteristics of HIV-TB co-infected patients who were included and excluded from the study due to missing data(N = 704).

Characteristics	Excluded from the study N(%)	Included in the study N (%)	p-value
**Age group (in years)**			
**0–14**	4 (4.0)	20(3.3)	0.9
**15–44**	76 (76.0)	478(79.1)	
**45–59**	18 (18.0)	91(15.1)	
	**Unfavorable**	**Favorable**	
**60 and above**	2 (2.0)	15(2.5)	
**Sex**			
**Male**	67 (67.0)	395(65.4)	0.8
**Female**	33 (33.0)	209(34.6)	
**Type of TB**			
**Smear positive**	62 (62.0)	305(50.5)	0.1
**Smear negative**	24 (24.0)	190(31.5)	
**Extrapulmonary**	14 (14.0)	109(18.0)	
**Total**	**100**	**604**	

TB = Tuberculosis; HIV = Human Immunodeficiency Virus.

**Table 2:** Characteristics of TB-HIV co-infected patients with favorable and unfavorable ART outcomes registered at 16 ART centres in Karnataka registered during Oct-Dec 2009(N = 604).

	Unfavorable	Favorable		
Characteristics	Outcome N(%)	Outcome N(%)	Total	p-value
	(n = 268)	(n = 336)		
**Age**				
**0–14**	8 (3.0)	12 (3.6)	20(3.3)	0.95
**15–44**	214 (79.9)	264 (78.5)	478(79.1)	
**45–59**	39 (14.5)	52 (15.5)	91(15.1)	
**60 and above**	7 (2.6)	8 (2.4)	15(2.5)	
**Sex**				
**Male**	197 (73.5)	198 (58.8)	395(65.4)	**<0.001**
**Female**	71 (26.5)	138 (41.2)	209(34.6)	
**Type of TB**				
**Smear positive**	145 (54.1)	161 (47.8)	306(50.5)	0.21
**Smear negative**	74 (27.6)	115 (34.3)	189(31.5)	
**Extra pulmonary**	49 (18.3)	60 (17.9)	109(18.0)	
**Category of TB treatment**				
**Cat 1**	232 (86.6)	291 (86.6)	523(86.6)	0.28
**Cat 2**	23 (8.6)	29 (8.6)	52(8.6)	
**Cat 3**	0 (0)	4 (1.2)	4(0.7)	
**Non-DOTS**	13 (4.8)	12 (3.6)	25(4.1)	
**Marital status**				
**Divorced**	7 (2.6)	10 (3.0)	17(2.8)	**0.05**
**Married**	183 (69.1)	228 (68.0)	411(68.3)	
**Unmarried**	40 (15.1)	32 (9.6)	72(12.2)	
**Widow/Widower**	35 (13.2)	65 (19.4)	100(16.7)	
**TB treatment outcome**				
**Favorable**	43 (16.3)	174 (52.4)	217(36.4)	**<0.001**
**Unfavorable**	220 (83.7)	157 (47.6)	377(63.6)	
**CD4 count at registration**				
**< 50**	44 (16.4)	38 (11.3)	82(13.6)	**0.06**
**50–200**	150 (56.0)	187 (55.5)	337(55.6)	
**200–350**	55 (20.5)	69 (20.6)	124(20.7)	
**>350**	19 (7.1)	42 (12.5)	61(10.1)	
**CD4 count at 12 months**				
**< 50**	4 (1.5)	10 (3.0)	14(2.3)	**<0.001**
**50–200**	19 (7.0)	50 (14.9)	69(11.4)	
**200–350**	31 (11.6)	82 (24.5)	113(18.7)	
**>350**	214 (79.9)	194 (57.6)	408(67.5)	
**Initiation of ART and ATT**				
**ART after ATT**	185 (69.0)	220 (65.4)	405(67.0)	0.18
**ART before ATT**	83 (31.0)	116 (34.6)	199(33.0)	

ART = Anti-Retroviral Therapy; TB = Tuberculosis; HIV = Human Immunodeficiency Virus; ATT = Anti-Tubercular Treatment

**Fig 1:**
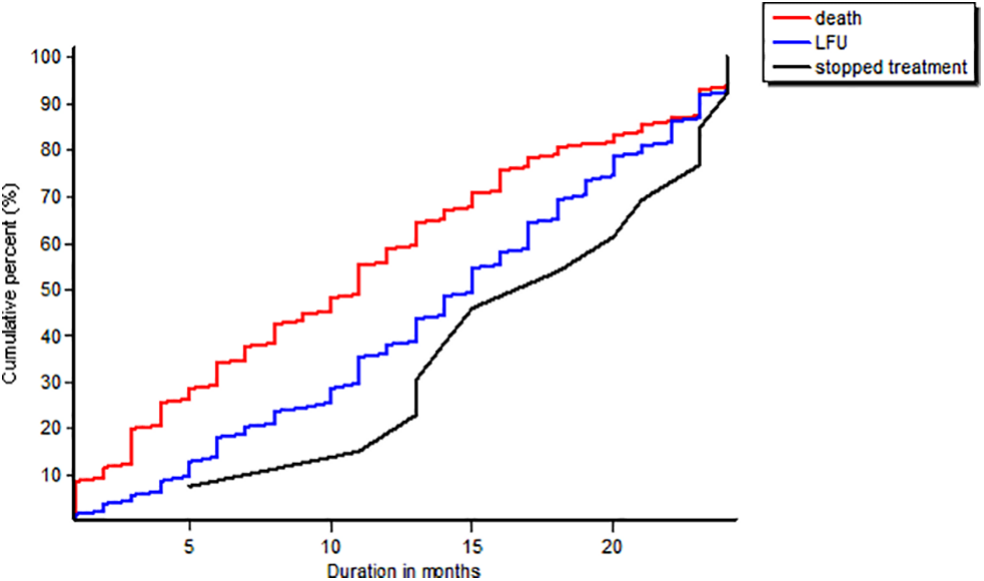
Cumulative plot showing the treatment outcomes of HIV-TB patients after two years of treatment initiation.

A total of 336 (55.6%) patients were alive and on ART after a two year follow up. At the end of first year, there were 65% deaths, 30% loss to follow-up and 15% had stopped treatment. **(Fig 1)**

Multivariate logistic regression showed that male sex (1.7, CI = 1.1–2.7), smear negative TB (2.0, CI = 1.2–3.2), CD4 count less than 50 cells per cumm (3.7, CI = 1.1–12.8) and unfavourable TB outcome had (6.1, CI = 3.9–9.8) significantly higher risk of unfavourable treatment outcome after a two year follow up. **(Table 3)**

**Table 3:** Adjusted OR from forward conditional multivariable logistic regression to determine factors associated with unfavourable treatment outcome among HIV-TB co-infected patients initiated on ART at 16 ART centres in Karnataka registered during Oct-Dec 2009.

Characteristics	Adjusted OR (95% CI)	p-value
**Male sex**	**1.7 (1.1–2.7)**	**0.02**
**Type of TB**		
Smear positive	1.5 (0.85–2.6)	0.16
Smear negative	**2.0 (1.2–3.2)**	**0.005**
Extra-pulmonary	Reference	Reference
**CD4 count at 12 months**		
<50	**3.7 (1.1–12.8)**	**0.03**
50–200	**2.6 (1.4–5.0)**	**0.004**
200–350	**2.4 (1.4–4.2)**	**0.002**
>350	Reference	Reference
**Unfavorable TB outcome**	**6.1 (3.9–9.8)**	**<0.001**

CI = Confidence Interval; OR = Odds Ratio; ART = Anti-Retroviral Therapy; TB = Tuberculosis; HIV = Human Immunodeficiency Virus

### Effects of time interval between the initiation of ART and TB treatment on all-cause mortality

**Table 4**shows the time interval between the initiation of ART and ATT. The variables age, sex, TB treatment outcome, type of TB, category of TB treatment and immunological response to ART is significantly associated with the time interval between the initiation of ART and TB treatment. More than double deaths were reported among patients where ART was started after ATT treatment compared to those who were on ART treatment before ATT. (**Table 5)** We evaluated the prognostic significance of the time interval between the initiation of ART and the initiation of TB therapy in cox proportional hazards analysis of all cause mortality. There were 140 deaths reported during 784 person years of followup [an incidence of 17.8 per 100 person years; 95% confidence interval (CI) 12.8–21.7]. Patients with early ART, delayed ART and TB diagnosed while on ART had a mortality risk of 19.3, 12.0 and 17.6 per 100 person years respectively. The interval between the initiation of ART and the initiation of TB therapy did not have a significant impact on all-cause mortality in the multivariate cox regression model. Smear negative TB and CD4 count less than 50 cells per cumm are significantly associated with all-cause mortality among HIV-TB co-infected patients. (**Table 6)**

**Table 4:** Time interval between the initiation of antiretroviral therapy (ART) and the initiation of tuberculosis (TB) treatment among HIV-TB co-infected patients (*n* = 602)*.

Time between initiation of ART and initiation of TB treatment (days)	N (%)
**ART before ATT**	191 (31.7)
0–60	97 (50.8)
61–180	27 (14.1)
181–365	34 (17.8)
>365	33 (17.3)
**ART after ATT**	411 (68.3)
0–60	288 (70.1)
61–180	81 (19.7)
181–365	38 (9.2)
>365	4 (1.0)

* timing of initiation of ART and ATT was missing for 2 patients

**Table 5:** Characteristics of patients with HIV-TB co-infection, by antiretroviral therapy (ART) initiation category.

Characteristics	ART before ATT	ART after ATT <60 days	ART after ATT >60 days	Total	p-value
**TB treatment outcome**					
Cured	55 (28.9)	70 (24.3)	34 (29.8)	159 (26.9)	0.01
On treatment	29 (15.3)	23 (8.0)	5 (4.4)	57 (9.6)	
Death	33 (17.4)	60 (20.8)	15 (13.2)	108 (18.2)	
Default	11 (5.8)	19 (6.6)	6 (5.3)	36 (6.1)	
Treatment complete	62 (32.6)	116 (40.3)	54 (47.4)	232 (39.2)	
Transferred out	0(0.0)	0 (0.0)	0 (0.0)	0 (0.0)	
Total	190	288	114	592	
**Sex**					
Male	132 (69.1)	195 (67.7)	69 (56.1)	396 (65.8)	0.04
Female	59 (30.9)	93 (32.3)	54 (43.9)	206 (34.2)	
Total	191	288	123	602	
**Age in years**					
0–14	0 (0.0)	11 (3.8)	9 (7.3)	20 (3.3)	0.002
15–44	158 (82.7)	229 (79.5)	89 (72.4)	476 (79.1)	
45–59	32 (16.8)	37 (12.8)	22 (17.9)	91 (15.1)	
>59	1 (0.5)	11 (3.8)	3 (2.4)	15 (2.5)	
Total	191	288	123	602	
**Type of TB**					
Smear positive	110 (57.6)	134 (46.5)	59 (48.0)	303 (50.3)	0.01
Smear negative	42 (22.0)	108 (37.5)	43 (35.0)	193 (32.1)	
Extra pulmonary	39 (20.4)	46 (16.0)	21 (17.1)	106 (17.6)	
Total	191	288	123	602	
**Category of TB treatment**					
Cat 1	155 (81.2)	267 (92.7)	98 (79.7)	520 (86.4)	0.002
Cat 2	23 (12.0)	14 (4.9)	16 (13.0)	53 (8.8)	
Cat 3	1 (0.5)	2 (0.7)	1 (0.8)	4 (0.7)	
Non-DOTS	12 (6.3)	5 (1.7)	8 (6.5)	25 (4.2)	
Total	191	288	123	602	
**Immunological response at 12 months after ART**					
CD4 count decreased <100	5 (4.6)	11 (8.3)	7 (9.9)	23 (7.4)	0.017
CD4 count decreased >100	3 (2.8)	5 (3.8)	9 (12.7)	17 (5.4)	
CD4 count increased	101 (92.7)	116 (87.9)	55 (77.5)	272 (87.2)	
Total	109	132	71	312	
**Treatment outcome at the end of two years**					
Alive and on ART	116 (60.4)	154 (53.1)	66 (54.1)	336 (55.6)	0.25
Death	42(21.9)	76(26.2)	22(18.0)	140(23.2)	
LFU	27(14.1)	39(13.5)	25(20.5)	91(15.1)	
Stopped treatment	2(1.0)	7(2.4)	4(3.3)	13(2.1)	
Transferred out	5(2.6)	14(4.8)	5(4.1)	24(4.0)	
Total	192	290	122	604	

**Table 6:** Cox proportional hazards analysis of all-cause mortality among HIV-TB co-infected patients.

Variable	Follow up (person-years)	Deaths	Unadjusted HR	p-value	Adjusted HR	p-value
**Sex**						
Male	584	102	0.9 (0.7–1.1)	0.13	0.9 (0.6–1.4)	0.65
Female	301	38	1.0		1.0	
**Time interval between ART and ATT initiation**						
ART before ATT	238	42	1.0		−	−
ART after ATT < 60 days	393	76	1.1 (0.7–1.6)	0.65	−	−
ART after ATT > 60 days	153	22	0.78 (0.4–1.3)	0.37	−	−
**Type of TB**						
Smear positive	379	81	1.3 (0.7–2.4)	0.4	1.3 (0.7–2.3)	0.4
Smear negative	272	40	**2.2 (1.2–3.8)**	**0.008**	**2.6 (1.5–4.8)**	**<0.001**
Extra-pulmonary	135	19	1.0		1.0	
**Age group in years**						
0–14	23	5	2.0 (0.5–8.2)	0.36	−	−
15–44	629	111	1.4 (0.4–4.4)	0.57	−	−
45–59	112	20	1.4 (0.4–4.8)	0.57	−	−
>59	21	4	1.0		−	−
**CD4 count at registration**						
CD4 count <50	116	36	**3.1 (1.4–7.1)**	**0.006**	**3.4 (2.2–6.4)**	**<0.001**
CD4 count 50–200	450	74	1.7 (0.8–3.7)	0.19	1.5 (0.7–3.4)	0.3
CD4 count 200–350	148	23	1.4 (0.6–3.3)	0.44	1.2 (0.5–2.9)	0.7
CD4 count >350	71	7	1.0			
**Marital status**						
Divorced	17	7	1.8 (0.7–4.8)	0.25	−	−
Married	541	94	1.0 (0.6–1.6)	0.88	−	−
Unmarried	99	16	1.1 (0.6–2.2)	0.71	−	−
Widow/Widower	121	21	1.0		−	−

ART = Anti-Retroviral Therapy; TB = Tuberculosis; HIV = Human Immunodeficiency Virus; ATT = Anti-Tubercular Treatment; HR = Hazards Ratio

## Discussion

We report for the first time, the ART treatment outcomes and their predictors in a large HIV-TB cohort in India.The results are of practical importance to a country like India which has 2.1 million HIV patients and accounts for highest TB burden in the world (23% of the world’s incident TB cases).[2][5] The study findings has following programmatic implications.

First, although the timing of initiation of ART was not associated with treatment outcome and mortality rate; the proportion of patients who died after two years was twice among patients where ART was started after ATT treatment compared to those who were on ART treatment before ATT. It perhaps indicates that there would have been a delay in the diagnosis of HIV and the patients could be reluctant to seek health care till they develop the symptoms of tuberculosis while being the late presenters to the health system. Currently, the TB services are mostly decentralized and integrated into the general health system while HIV services remain largely centralized. The gap in infrastructure between the TB and HIV programme might have led to suboptimal linkages.

Second, HIV-TB patient with unfavorable TB outcome has six times risk of having an unfavorable ART outcome and nine times risk for all-cause mortality at the end of two years. It was also significantly associated with the time interval between initiation of ART and ATT. In biological perspective, development of TB is associated with increased HIV-1 replication. Both HIV-1 load and heterogeneity appear to be affected by tuberculosis infection.[6] Unfavorable TB outcome leads to persistence of TB infection for a longer period in the body leading to poorer outcomes. CD4 count less than 50 cells per cumm has 3–4 times risk of unfavorable outcome and poorest survival probability after two years. It is significantly associated with all-cause mortality among HIV-TB co-infected patients which is consistent with other studies.[7][8][9][10] Shafer et al has demonstrated that CD4 count was the only independent predictor of survival among HIV-TB co-infected patients.[11] Smear negative TB was also associated with mortality among HIV-TB co-infected patients in contrast to other studies which showed either no association or association of smear positive or extra-pulmonary TB with mortality.[8][9] The association to smear negative TB could be attributed to late presentation to the health system that may unmask the smear positive signs and symptoms.

We presume the shortcomings can be addressed in following ways (a) intensifying community awareness regarding early health care seeking behavior among people with symptoms of HIV/AIDS and TB. (b) accessibility issues in terms of diagnosis and treatment have to be addressed which might prevent patients presenting ‘too late’. Mechanisms should be in place to provide access to HIV testing and ART services at the TB treatment sites itself to avoid delay in ART initiation. (c) appropriate newer TB diagnostic tools to be made to reduce the diagnostic and treatment delays linked to smear negative pulmonary TB and through simpler and more efficient TB diagnostic tools for use in resource limited settings. Even for smear positive TB and HIV co-infected patients sputum smear microscopy is not a sensitive tool to diagnose TB and the HIV patients should be provided an access to a culture based diagnosis preferably liquid culture technology or equivalent technology. (d) the health care settings frequented by high numbers of HIV-infected persons, it becomes necessary to implement airborne infection control measures. Simple administrative and environmental measures aimed at reducing exposure of HIV-infected patients to M. tuberculosis bacilli will be worthwhile.(e) WHO recommended isoniazid preventive therapy (IPT) should be implemented as a policy for prevention of incident TB among HIV infected patients. These above measures may decrease the transmission of TB and help the programme to ensure timely detection of TB and initiation of ATT.

Third, earlier trials have shown that early ART initiation (less than 2 weeks of ATT) improved survival in HIV-TB co-infected patients.[12][13][14] However, in our study the overall mortality of patients with TB in whom ART was initiated early was not significantly different from those patients with delayed ART initiation. Our findings are also consistent with another large observational database which reported a non-significant impact of delayed ART initiation among HIV-TB co-infected patients on mortality.[4] The primary endpoint of the global STRIDE study demonstrated the benefit of early ART initiation among patients with CD4 cell counts less than 50 cells per cumm, however the primary endpoint was new AIDS-defining illness or death.[12]

Operationally, we have defined early ART with a cut-off of 60 days, although previous studies have used different cut-offs such as 90 days.[4] The comparison of outcomes between 60 and 90 days, were similar with regard to the hazard ratios for death (data not shown).

Fourth, among those who were initiated on ART before starting on ATT, a significant proportion of patients (51%) had ART started within 60 days before ATT initiation. Such cases could be due to unmasking of latent TB i.e., TB-IRIS. Luetkemeyer et al. demonstrated increased risk of TB IRIS among those with very low CD4 cell counts of less than 50 cells per cumm thus necessitating screening thoroughly for TB before starting ART among patients with low CD4 count. [15]

Fifth, an important assumption of survival analysis is that the censored patients are considered to have survival prospects similar to the participants who continued to be followed up. We found that the patients who were lost to follow up were similar (p>0.05) to those who could be followed up in terms of their CD4 count, type of TB, category of TB and timing of initiation of ATT and ART which are the possible factors determining treatment outcomes as reported in this study. (data not shown) Thus, we can confidently opine that the participants who were drop out in the study did so due to reasons unrelated to the study and had similar survival prospects compared to those who could be followed up and thus satisfies the assumption of non-informative censoring.

The strengths of this study is that a large cohort of HIV-TB co-infected patients were studied, deaths were ascertained, the loss to follow up was minimal and the data was from a programme setting which reflected the operational reality on the ground.The following were the limitations of the study (a) observational nature of the data and the retrospective collection of data (b) other associated co-morbid factors for death in patients were not studied (c) the average CD4 count of cohort at different points of time were taken into consideration as the CD4 cell counts for quite a number of patients were not done or not recorded (d) only those TB-HIV co-infected patients who were registered at the ART centers were studied; there would have been a number of HIV-TB co-infected patients who were diagnosed and did not access the ART services during this period. (e) there is ample scope in this study to follow up the cohort of patients for next two years as there is sparse evidence of such findings under programmatic settings.

## Conclusion

The programmes needs to urgently review the existing strategies and strengthen HIV-TB collaborative activities for timely treatment initiation with intensive monitoring of HIV-TB patients on treatment.

## References

[1] Sharma SK, Mohan A, Kadhiravan T. HIV-TB co-infection: Epidemiology, diagnosis & management. Indian J Med Res 2005;121:550–67 15817963

[2] TB India 2012. Revised National Tuberculosis Control Programme: Annual Status Report. Central TB Division, Ministry of Health and Family Welfare: New Delhi; 2012. Available: http://tbcindia.nic.in/pdfs/TB%20India%202012-%20Annual%20Report.pdf. Accessed 26 December 2014.

[3] TB India 2013. Revised National Tuberculosis Control Programme: Annual Status Report. Central TB Division, Ministry of Health and Family Welfare: New Delhi; 2013. Available: http://www.tbcindia.nic.in/pdfs/TB%20India%202013.pdf. Accessed 26 December 2014.

[4] Han SH, Zhou J, Lee MP, Zhao H, Chen YMA, Kumarasamy N, et al Prognostic Significance of the Interval Between the Initiation of Antiretroviral Therapy and the Initiation of Anti-tuberculosis Treatment in HIV/Tuberculosis co-infected patients. HIV Medicine 2014;15(2):77–85 10.1111/hiv.12073 23980589 PMC3947330

[5] Department of AIDS Control, Ministry of Health and Family Welfare New Delhi. Technical Report India 2012: HIV estimates-2012; 2012. Available: http://www.naco.gov.in/upload/Surveillance/Reports%20&%20Publication/Technical%20Report%20-%20India%20HIV%20Estimates%202012.pdf.

[6] Toossi Z. Virological and immunological impact of tuberculosis on human immunodeficiency virus type 1 disease. J Infect Dis 2003;188:1146–55. 14551885 10.1086/378676

[7] MacPherson P, Dimairo M, Bandason T, Zezai A, Munyati SS, Butterworth AE, et al Risk factors for mortality in smear-negative tuberculosis suspects: a cohort study in Harare, Zimbabwe. Int J Tuberc Lung Dis 2011, 15(10):1390–1396 10.5588/ijtld.11.0056 22283900 PMC3272461

[8] Sileshi B, Deyessa N, Girma B, Melese M, Suarez P. Predictors of mortality among TB-HIV Co-infected patients being treated for tuberculosis in Northwest Ethiopia: a retrospective cohort study. BMC Infectious Diseases 2013 13:297 10.1186/1471-2334-13-297 23815342 PMC3703293

[9] Ismail I, Bulgiba A (2013) Predictors of Death during Tuberculosis Treatment in TB/HIV Co-Infected Patients in Malaysia. PLoS ONE 8(8):e73250 10.1371/journal.pone.0073250 23951346 PMC3741191

[10] Català L, Orcau A, García de Olalla P, Millet JP, Rodríguez-Mondragón A, Caylà JA, et al (2011) Survival of a large cohort of HIV-infected tuberculosis patients in the era of highly active antiretroviral treatment. Int J Tuberc Lung Dis 15: 263–269. . 21219692

[11] Shafer RW, Bloch AB, Larkin C, Vasudavan V, Seligman S, Dehovitz JD, et al Predictors of survival in HIV-infected tuberculosis patients. AIDS 1996;10(3):269–72 8882666 10.1097/00002030-199603000-00005

[12] Havlir DV, Kendall MA, Ive P, Kumwenda J, Swindells S, Qasba SS, et al Timing of antiretroviral therapy for HIV1 infection and tuberculosis. N Engl J Med 2011;365:1482–91. 10.1056/NEJMoa1013607 22010914 PMC3327101

[13] Blanc FX, Sok T, Laureillard D, Borand L, Rekacewicz C, Nerrienet E, et al Earlier versus later start of antiretroviral therapy in HIVinfected adults with tuberculosis. N Engl J Med 2011; 365: 1471–1481. 22010913 10.1056/NEJMoa1013911 PMC4879711

[14] Abdool Karim Q, Abdool Karim SS, Baxter C, Friedland G, Gengiah T, Gray A, et al The SAPIT trial provides essential evidence on risks and benefits of integrated and sequential treatment of HIV and tuberculosis. S Afr Med J 2010; 100: 808–809. 21414268 10.7196/samj.4621

[15] Luetkemeyer AF, Kendall MA, Nyirenda M, Wu X, Ive P, Benson CA, et al Tuberculosis immune reconstitution inflammatory syndrome in A5221 STRIDE: timing, severity, and implications for HIV-TB programs. J Acquir Immune DeficSyndr. 2014;65(4):423–8. 10.1097/QAI.0000000000000030 PMC3943693 24226057

